# Regional tau burden tracks with lower microglial HLA class II transcription in the human brain

**DOI:** 10.3389/fnins.2026.1979981

**Published:** 2026-10-07

**Authors:** Fan Li, Suqin Jin, Ping Wang, Hui Yang, Xianghua Zhuang, Feifei Feng, Peng Cheng

**Affiliations:** 1 Department of Neurology, The Second Qilu Hospital of Shandong University, Cheeloo College of Medicine, Shandong University, Jinan, Shandong, China; 2 Department of Endocrinology, The Second Qilu Hospital of Shandong University, Cheeloo College of Medicine, Shandong University, Jinan, Shandong, China; 3 Department of Pulmonary and Critical Care Medicine, The Second Qilu Hospital of Shandong University, Cheeloo College of Medicine, Shandong University, Jinan, Shandong, China

**Keywords:** Alzheimer’s disease, antigen presentation, HLA class II, iPSC-microglia, microglia, postmortem brain, single-nucleus RNA sequencing, tau

## Abstract

**Background:**

Human leukocyte antigen class II (HLA-II) genes mark several microglial states in Alzheimer’s disease, but it remains unclear how their average transcription relates to tau measured in the same brain region.

**Methods:**

We used a locked 13-gene HLA-II panel selected by biological function, cross-cohort detection, and variance, without considering effect estimates. The primary analysis was conducted using the Seattle Alzheimer’s Disease Brain Cell Atlas (SEA-AD): 361 donor-region observations from 79 donors across 6 cortical regions were modeled with within-donor pathology decomposition, region fixed effects, and a donor random intercept. A paired entorhinal-versus-somatosensory analysis in GSE160936 (11 donors) provided additional support for our findings. Sensitivity analyses addressed amyloid-β, RNA quality, neuronal-nuclei enrichment, microglial-state composition, published state annotations, alternative panels, region omission, and assay modality. Public human induced pluripotent stem cell (iPSC)-microglia data were used to provide a distinct tau-exposure context.

**Results:**

In the SEA-AD study, a higher local AT8-positive area within a donor was associated with a lower expanded HLA-II score (*β* = −0.174, 95% confidence interval [CI]: −0.297 to −0.052; *p* = 0.005). The estimate remained negative after adjusting for local amyloid-β, RNA integrity number, and the NeuN-positive fluorescence-activated nuclei sorting (FANS) fraction (*β* = −0.167, 95% CI: −0.289 to −0.045; *p* = 0.007). This result was consistent after adjusting for the proportion of a published Damage-Associated Microglia (DAM)-enriched state. A formal tau-minus-amyloid-β contrast was not significant. In GSE160936, the expanded-panel pTau coefficient was negative (*β* = −0.533); however, the primary exact test was not significant after the two-test correction (*p* = 0.083; *q* = 0.166). Fibrillar tau exposure lowered the score derived from the 12 detectable panel genes in iPSC-microglia (difference = −1.343; exact *p* = 0.029).

**Conclusion:**

In a larger multi-region cohort and a smaller paired cohort, we observed that regional tau burden was associated with lower levels of pooled microglial HLA-II transcription. The association was not explained solely by the measured proportion of the DAM-like state. However, the data do not establish tau specificity, direct suppression, protein-level antigen presentation, or a causal mechanism.

## Introduction

Microglia in Alzheimer’s disease (AD) occupy multiple, partly overlapping transcriptional states rather than a single activation phenotype. Human single-cell and spatial studies have described human leukocyte antigen class II (HLA-II) and antigen-processing programs alongside lipid, complement, interferon, stress, and homeostatic responses (Mathys et al., 2019; Prater et al., 2023; Gabitto et al., 2024; Mathys et al., 2024; Mancuso et al., 2024; Tuddenham et al., 2024; Martins-Ferreira et al., 2025). Human genetic and functional evidence also implicates HLA-DQ and HLA-DR genes (Afify et al., 2024; Mary et al., 2024; Capitani and Carpanini, 2025).

The spatial behavior of these programs is context-dependent. HLA-II-rich myeloid cells accumulate near plaques and mixed lesions across several datasets, while other studies report reduced HLA-related expression in selected tau-associated microenvironments (Kwon et al., 2023; Wachter et al., 2024; Miyoshi et al., 2024; Avey et al., 2025; Fixemer et al., 2025; Mrdjen et al., 2025; Sanchez-Molina et al., 2026). Lu et al. (2026) further separated an early, plaque-induced program from a later antigen-presentation-rich program at the amyloid-β (Aβ)-tau transition. These local findings do not determine how the average HLA-II program across eligible microglia behaves as regional tau burden increases.

Regional comparisons require donor-aware inference because nuclei and regions from one person are not independent biological replicates (Crowell et al., 2020; Squair et al., 2021; Zimmerman et al., 2021). They also require an explicit identity rule because resident microglia share immune transcripts with perivascular and border-associated macrophages (Masuda et al., 2019; Sankowski et al., 2019; Van Hove et al., 2019; Silvin et al., 2022; Paolicelli et al., 2022). We therefore maintained the donor as the biological unit, defined resident-compatible cells without using HLA-II outcome genes, and treated state composition as a competing explanation rather than an afterthought.

We investigated whether a brain region with higher tau levels compared to another region from the same donor also had higher or lower microglial HLA-II transcription. The Seattle Alzheimer’s Disease Brain Cell Atlas (SEA-AD) provided data for the primary multiregion analysis; the paired entorhinal and somatosensory cortex (SSC) from GSE160936 provided a small supportive analysis (Smith et al., 2022; Gabitto et al., 2024). We then tested panel choice, Aβ levels, local technical and tissue covariates, published microglial states, and assay modality. We also examined a separate human induced pluripotent stem cell (iPSC)-microglia tau-exposure dataset (Karabova et al., 2026).

## Materials and methods

### Study design and evidence roles

We performed a secondary analysis of de-identified public data (Figures 1A,C,D; Table 1). The SEA-AD was designated as the primary postmortem cohort because it contained 79 donors, 6 cortical regions, continuous region-matched AT8 pathology, published cell states, and sample-level covariates (Gabitto et al., 2024). GSE160936 supplied a supportive paired-region analysis of the entorhinal cortex (EC) and somatosensory cortex (SSC) from 11 complete donors (Smith et al., 2022). The iPSC-microglia study served as an orthogonal perturbation context, not as mechanistic validation (Karabova et al., 2026). Cohort estimates were not pooled due to differences in pathology assays and sampling designs.

**Figure 1:**
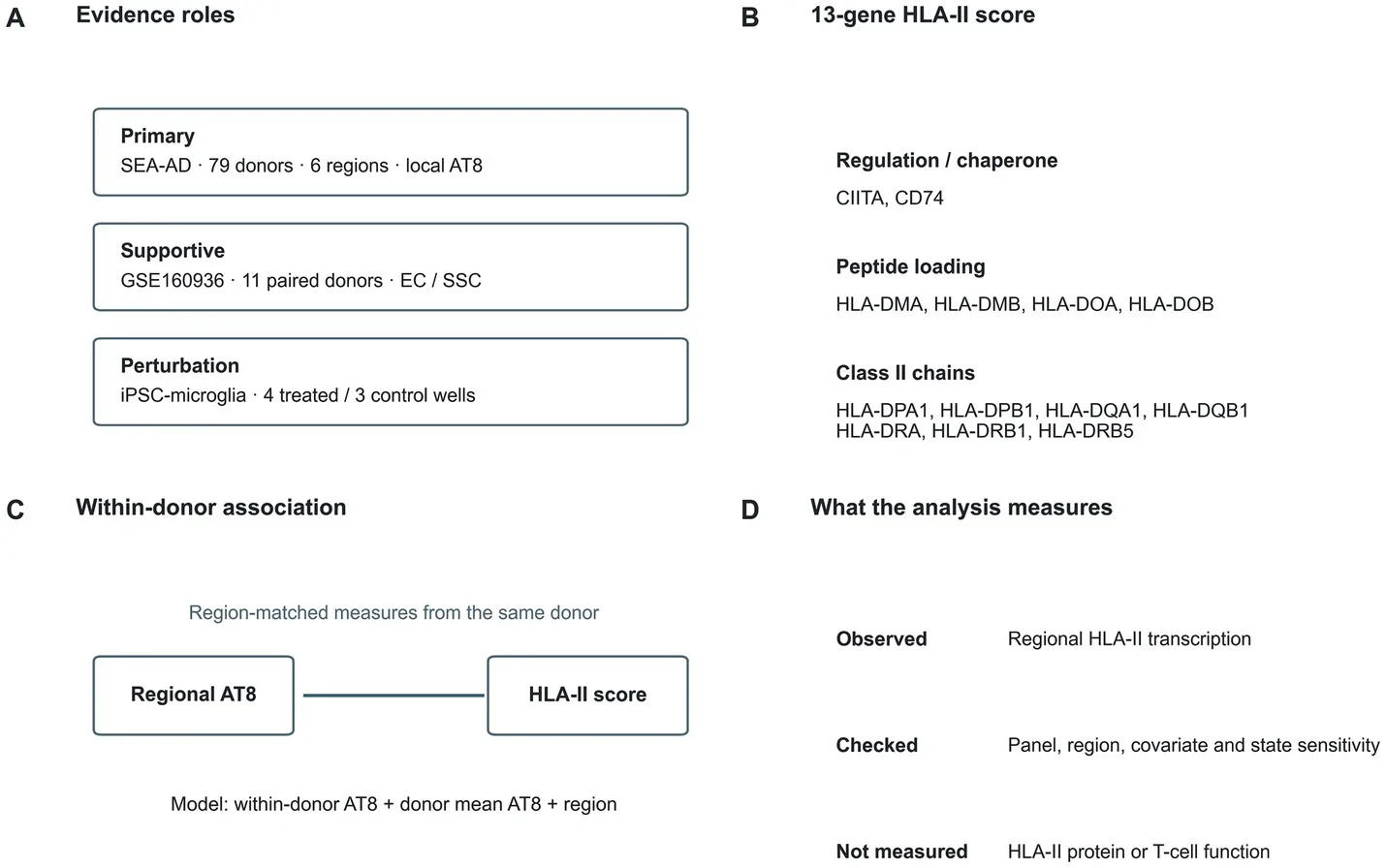
Study design and inferential boundaries. **(A)** Evidence roles were fixed by sample size and design. **(B)** The 13-gene HLA-II panel was locked before the revision models were rerun. **(C)** The primary coefficient relates a region’s deviation from the donor’s mean pathology to its HLA-II score while modeling the donor mean and the region. **(D)** This study measures transcriptional association, not protein-level antigen presentation or T-cell function. EC, entorhinal cortex; SSC, somatosensory cortex.

**Table 1:** Dataset roles and inferential units.

Dataset	Role	Biological units	Regions/comparison	Primary use
SEA-AD singleome	Primary	79 donors; 361 donor-regions	Six cortical regions	Within-donor local AT8 model; covariate and state analyses
GSE160936	Supportive	11 complete paired donors	EC versus SSC	Exact paired regional contrast
SEA-AD multiome	Modality sensitivity	14 overlapping donors; 42 donor-regions	Three cortical regions	Assay sensitivity; not independent replication
Karabova iPSC-microglia	Perturbation context	4 treated and 3 control wells per contrast	Tau exposure	Transcriptional response; not mechanistic validation

### Locked HLA-II panel and scoring

The revision analysis contract was finalized on 14 September 2026 before rerunning any outcome model. A total of 13 candidate genes captured HLA-II regulation, invariant-chain/chaperone function, peptide loading, and HLA-DP, HLA-DQ, and HLA-DR chains: cluster of differentiation 74 (CD74), class II major histocompatibility complex transactivator (CIITA), human leukocyte antigen-DM alpha (HLA-DMA), human leukocyte antigen-DM beta (HLA-DMB), human leukocyte antigen-DO alpha (HLA-DOA), human leukocyte antigen-DO beta (HLA-DOB), human leukocyte antigen-DP alpha 1 (HLA-DPA1), human leukocyte antigen-DP beta 1 (HLA-DPB1), human leukocyte antigen-DQ alpha 1 (HLA-DQA1), human leukocyte antigen-DQ beta 1 (HLA-DQB1), human leukocyte antigen-DR alpha (HLA-DRA), human leukocyte antigen-DR beta 1 (HLA-DRB1), and human leukocyte antigen-DR beta 5 (HLA-DRB5) (Figure 1B). Eligibility criteria required a unique feature in both postmortem objects, non-zero variance, and detection in at least 10% of compatible resident-compatible pseudobulks in each cohort. No effect estimate, *p*-value, or pathology direction entered selection. All 13 genes passed. Within each analytic object, log-expression for each gene was standardized across eligible donor-region units and averaged with equal weight. The submitted six-gene score and the seven HLA-II genes in the Lu late plaque-induced gene set were retained as sensitivity panels. HLA-DRB5 was unavailable in the iPSC normalized-count matrix, so the analysis used the 12 detectable genes. Full eligibility and coverage are in Supplementary Table S1, Figure S1.

### Cell identity and DAM-like context

Resident-compatible populations were defined using lineage information independent of the HLA-II panel. Perivascular or border-associated macrophage markers (including Mannose Receptor C-Type 1 [MRC1], Lymphatic Vessel Endothelial Hyaluronan Receptor 1 [LYVE1], Coagulation Factor XIII A Chain [F13A1], CD163, Folate Receptor Beta (FOLR2), and Macrophage Activating Factor [MAF]) and monocyte-like identities informed exclusions; disease-associated activation alone did not (Chhatbar et al., 2026). To avoid circularity, the Damage-Associated Microglia (DAM)-like context was summarized with Apolipoprotein E (APOE), C-Type Lectin Domain Family 7 Member A (CLEC7A), Cystatin F (encoded by the *CST7* gene) (CST7), Cathepsin B (CTSB), Cathepsin D (CTSD), Glycoprotein Nonmetastatic Melanoma Protein B (GPNMB), Lipoprotein Lipase (LPL), Secreted Phosphoprotein 1 (SPP1), and Triggering Receptor Expressed on Myeloid Cells 2 (TREM2), explicitly excluding HLA-II genes (Keren-Shaul et al., 2017; Krasemann et al., 2017). In SEA-AD, Micro-PVM_3-SEAAD was the prespecified published DAM-enriched state. We modeled its proportion, adjusted the pooled HLA-II model for it, and estimated HLA-II associations within published resident-compatible states with at least 20 donors. Micro-PVM_4-SEAAD, the rare antigen-presenting state, did not meet that donor threshold.

### SEA-AD primary model

The primary analysis included 361 resident-compatible donor-region pseudobulks from 79 donors across the dorsolateral prefrontal cortex (DFC), the inferior temporal gyrus (ITG), the medial entorhinal cortex (MEC), the middle temporal gyrus (MTG), the superior temporal gyrus (STG), and the primary visual cortex (V1C). Donors contributed at least three eligible regions. Local log1p-transformed percent AT8-positive area was decomposed into the donor mean and each region’s deviation from that mean (Wang and Maxwell, 2015). The mixed model was HLA-II score = within-donor AT8 + donor-mean AT8 + region fixed effects + donor random intercept. Thus, the within-donor coefficient asks whether a region with more tau than that donor’s sampled-region average also has a different HLA-II score. We repeated the inference with donor-clustered standard errors and 2,000 whole-donor bootstrap samples and omitted each region in turn.

### SEA-AD pathology, covariate, state, and modality analyses

A joint model entered the within- and between-donor components of local AT8 and local Aβ. The prespecified coefficient contrast tested AT8 minus Aβ; the significance of one coefficient and not the other was not treated as evidence of a difference. The covariate model additionally included RNA integrity number (RIN) and the NeuN-positive fluorescence-activated nuclei sorting (FANS) fraction. State composition and within-state analyses followed the rules above. Multiome pseudobulks were analyzed separately. All 41 multiome donors overlapped with the singleome set; 14 of them contributed to the three regions required for the model. The result was therefore considered assay-modality sensitivity rather than independent biological replication.

### GSE160936 paired supportive analysis

For the 11 donors with complete, resident-compatible EC and SSC summaries, EC-minus-SSC differences were calculated for the HLA-II score and the region-matched pTau-positive-cell percentage or the Aβ-positive area. SSC had substantially less pTau than EC (median 0.052% versus 0.353%; Figure 2D). Standardized score differences were regressed on standardized pathology differences. The primary small-sample test enumerated all 2^11^ sign flips of the paired regional contrast. Benjamini-Hochberg correction of the two primary exact *p*-values was performed separately within each panel. Heteroscedasticity-Consistent Standard Error Estimator Version 3 (HC3) intervals, Theil-Sen slopes, leave-one-donor-out estimates, Cook’s distance, and Difference in Beta (DFBETA) were descriptive robustness measures. DAM-like cluster fraction and Aβ were examined in joint sensitivity models.

**Figure 2:**
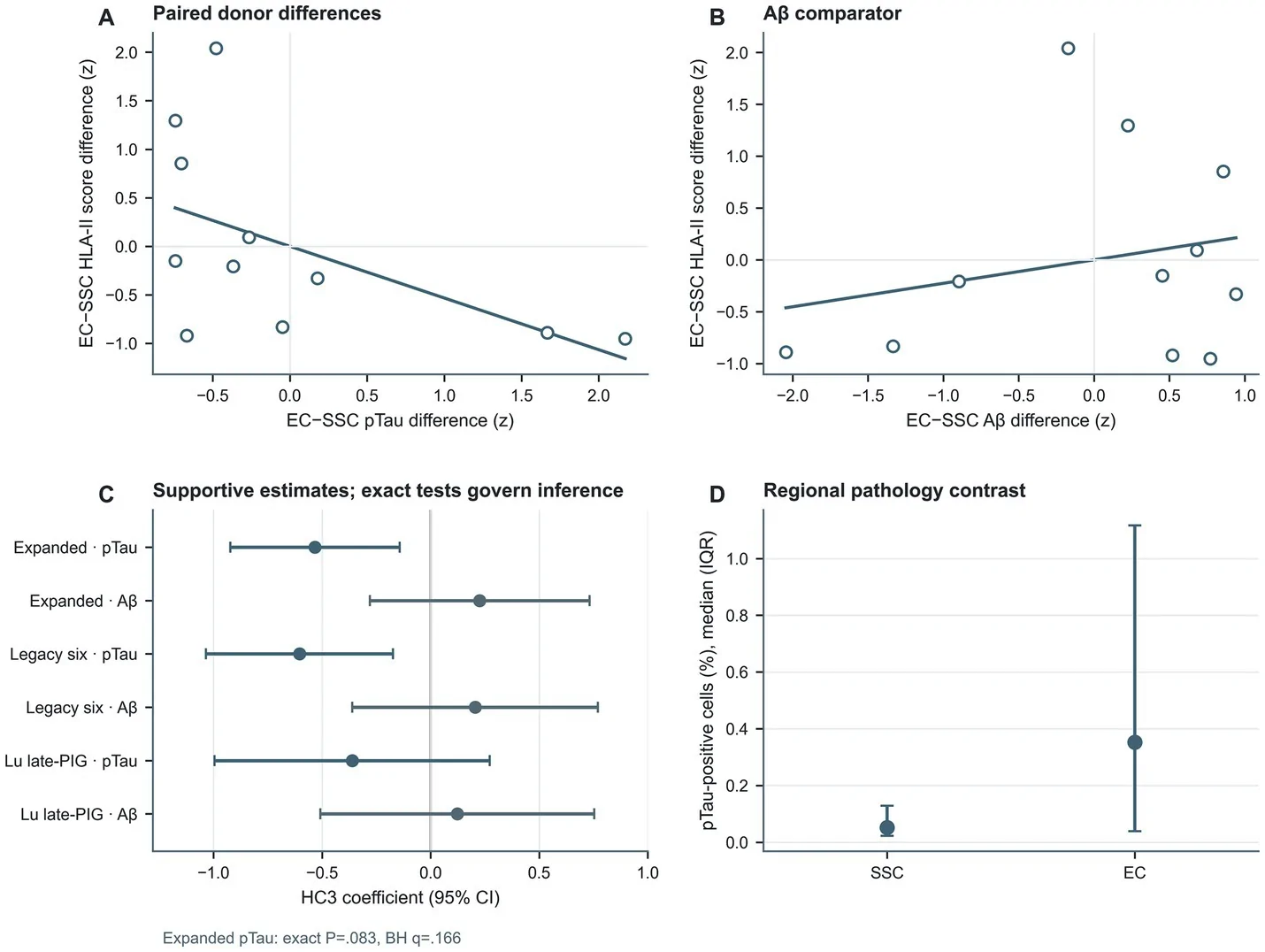
Supportive paired analysis in GSE160936. **(A, B)** Individual-donor EC-minus-SSC differences for the expanded HLA-II score versus region-matched pTau or Aβ. **(C)** HC3 coefficients and 95% confidence intervals across panels; exact sign-flip tests, not HC3 *p*-values, governed small-sample inference. The expanded-panel pTau result had an exact *p* = 0.083 and BH *q* = 0.166. **(D)** Regional pTau distributions show a lower burden in the SSC than in the EC. Aβ, amyloid-β; EC, entorhinal cortex; pTau, phosphorylated tau; SSC, somatosensory cortex.

### Human iPSC-microglia tau exposure

Normalized sample-level counts and published DESeq2 outputs were obtained from human iPSC-microglia exposed to recombinant fibrillar tau, recombinant monomeric tau, vehicle, human AD-derived tau, or tau-depleted material (Karabova et al., 2026). For each contrast, genes were standardized across the seven wells and averaged. The treatment-minus-control difference was tested by enumerating all 35 allocations of four treated and three control wells. The fibrillar tau versus vehicle contrast was prespecified as the primary contrast; the monomeric tau and human AD tau contrasts were contextual. All well values are shown (Figure 3A), and gene-level published fold changes are reported (Figure 3C). General stress, uptake, toxicity, or preparation effects cannot be separated from a tau-responsive transcriptional change (Figure 3D).

**Figure 3:**
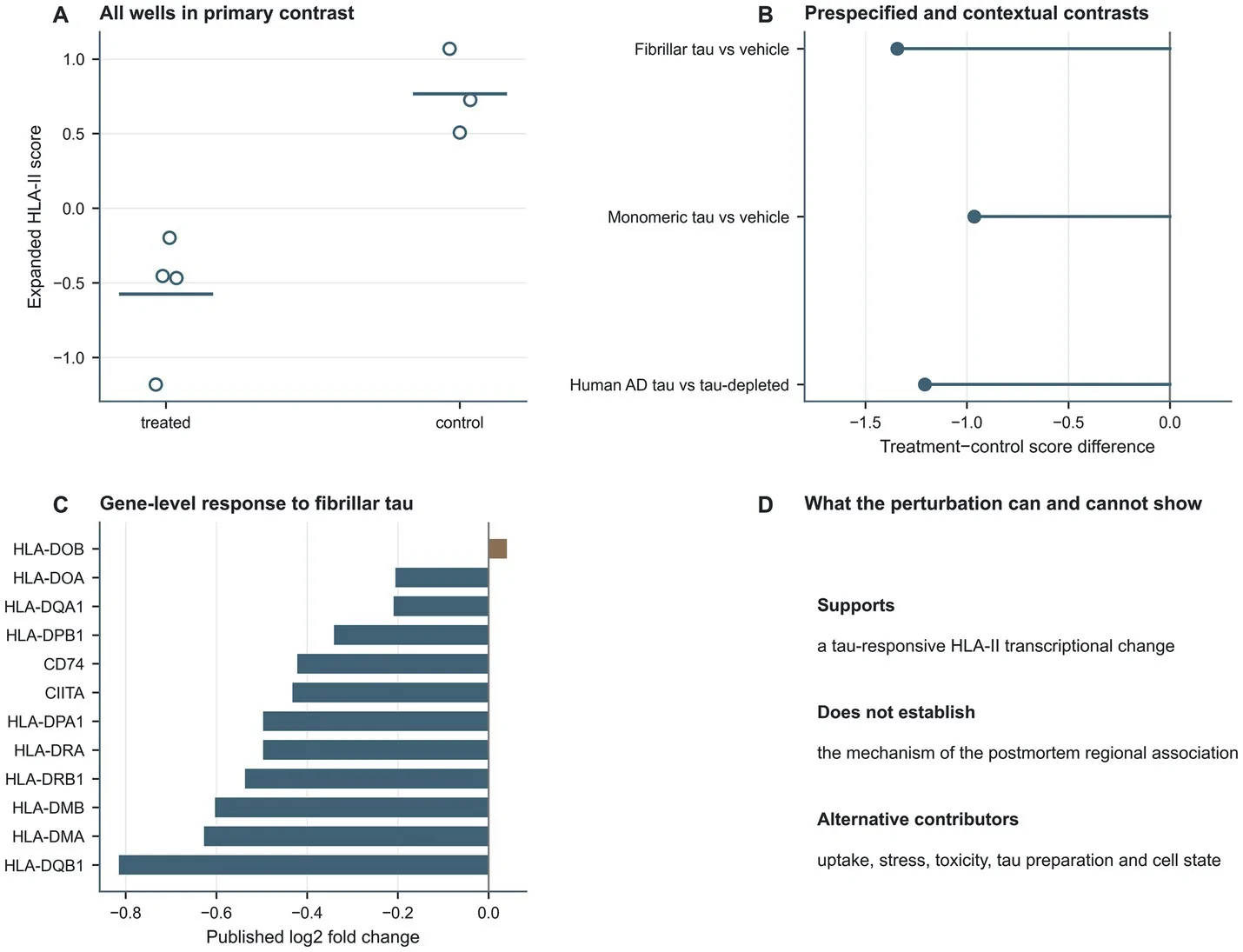
HLA-II transcription after tau exposure in human iPSC-microglia. **(A)** All four treated and three control well scores for fibrillar tau versus vehicle; horizontal bars denote means. **(B)** Treatment-minus-control score differences for the prespecified fibrillar-tau contrast and two contextual contrasts; line segments connect zero to each estimate and are not confidence intervals. **(C)** Published gene-level log2 fold changes for fibrillar tau versus vehicle among the 12 detectable panel genes. **(D)** Interpretation of the perturbation result. AD, Alzheimer’s disease; iPSC, induced pluripotent stem cell.

### Statistical principles

Postmortem donors and iPSC wells were the independent biological units for their respective analyses. Nuclei, cells, regions, and assay profiles were nested observations and were not counted as additional donors. Tests were two-sided. Exact *p*-values governed the small paired cohort; mixed-model and donor-bootstrap estimates governed SEA-AD. The cross-sectional postmortem coefficients are associations, not causal effects. Analyses were performed in Python 3.12 using pandas, NumPy, SciPy, statsmodels, and scikit-learn; the code, the locked contract, aggregate outputs, and file hashes are archived with the reproducibility record.

## Results

### The expanded panel remained inversely associated with local tau in SEA-AD

All 13 candidate genes passed the locked detection and variance rules (Supplementary Table S1). In the primary SEA-AD model, a higher within-donor local AT8 burden was associated with a lower expanded HLA-II score (*β* = −0.174, 95% confidence interval (CI): −0.297 to −0.052; *p* = 0.005; Figure 4A). The legacy six-gene and Lu late-PIG HLA-II panels produced nearly identical estimates (*β* = −0.189 for each; Figure 4A). Leave-one-region-out estimates remained negative, ranging from −0.318 to −0.099, although intervals crossed zero when DFC or MTG was omitted (Figure 4B). Regional AT8 burden was lowest in V1C and highest in MEC, with overlapping distributions elsewhere (Figure 4D; Supplementary Figure S3).

**Figure 4:**
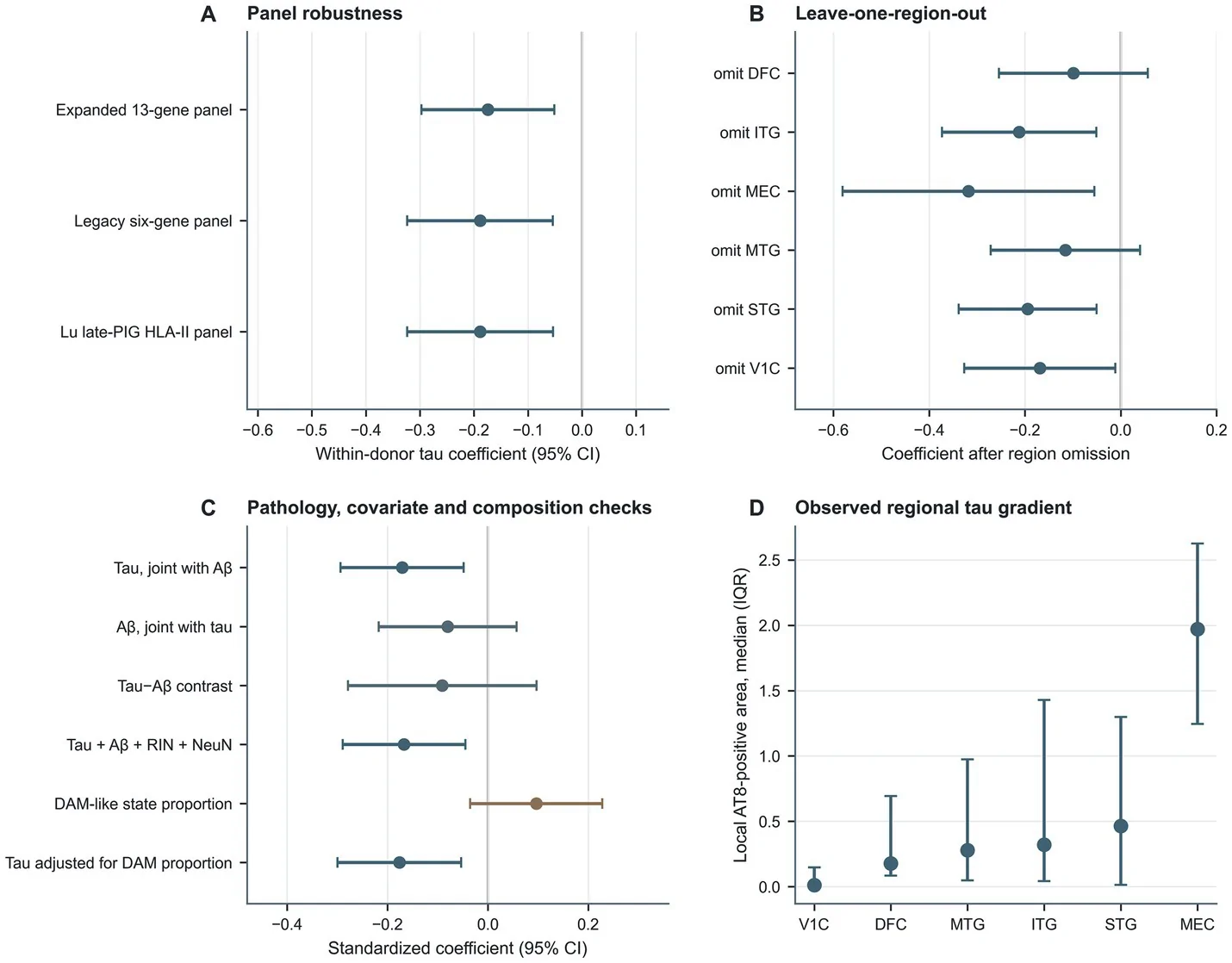
Primary SEA-AD analyses. **(A)** Within-donor local AT8 coefficients for the expanded, legacy six-gene, and Lu late-PIG HLA-II panels. **(B)** Expanded-panel coefficients after omitting each region. **(C)** Joint pathology, regional covariate, and DAM-state-composition analyses. The tau-minus-Aβ contrast was not significant. **(D)** Median and interquartile range of local AT8-positive area across regions. Points and intervals in **A–C** are mixed-model estimates and 95% confidence intervals. Aβ, amyloid-β; DFC, dorsolateral prefrontal cortex; ITG, inferior temporal gyrus; MEC, medial entorhinal cortex; MTG, middle temporal gyrus; STG, superior temporal gyrus; V1C, primary visual cortex.

### The association survived measured regional covariates but did not establish tau specificity

When local AT8 and Aβ were entered together, the AT8 coefficient remained negative (*β* = −0.171, 95% CI: −0.294 to −0.048; *p* = 0.006), whereas the Aβ coefficient was −0.080 (95% CI: −0.217 to 0.057; *p* = 0.253; Figure 4C). The formal AT8-minus-Aβ contrast was not significant (difference = −0.091, 95% CI: −0.279 to 0.097; *p* = 0.343); we therefore describe the pattern as more consistent for tau, not tau-specific. Additional adjustment for local Aβ, RIN, and NeuN-positive FANS fraction yielded *β* = −0.167 (95% CI: −0.289 to −0.045; *p* = 0.007; Figure 4C; Supplementary Table S2).

### Measured DAM-like composition did not account for the pooled association

The proportion of the published DAM-enriched Micro-PVM_3-SEAAD state was not significantly associated with within-donor AT8 (*β* = 0.097, 95% CI: −0.035 to 0.228; *p* = 0.151). Adding that proportion to the pooled model left the AT8 coefficient essentially unchanged (*β* = −0.176, 95% CI: −0.300 to −0.053; *p* = 0.005; Figure 4C). Within-state estimates were negative in Micro-PVM_2 (*β* = −0.139, *p* = 0.035), Micro-PVM_2_3-SEAAD (*β* = −0.042, *p* = 0.507), and Micro-PVM_3-SEAAD (*β* = −0.138, *p* = 0.123; Supplementary Figure S1, Table S3). Micro-PVM_4-SEAAD was too sparse for the prespecified model. These results argue against a single measured composition shift as the sole explanation, but they do not identify a uniform within-state mechanism.

### The small paired cohort supported direction, not statistical confirmation

In GSE160936, the expanded-panel coefficient for EC-minus-SSC pTau was −0.533 (HC3 95% CI: −0.923 to −0.143; Figures 2A,C). The exact sign-flip *p*-value was 0.083, and the two-test BH q-value was 0.166; this result was therefore not statistically significant under the prespecified small-sample procedure. The Aβ coefficient was 0.226 (exact *p* = 0.415; *q* = 0.415; Figures 2B,C). Leave-one-donor-out pTau coefficients ranged from −0.634 to −0.436, and the Theil-Sen slope was −0.396. The largest Cook’s distance was 0.291 (donor A282/14), below the common 4/n reference of 0.364 (Supplementary Figure S2, Table S4). The legacy six-gene exact *p*-value was 0.042, but its corrected q was 0.084. Accordingly, this cohort provides directionally consistent but uncertain support.

### Multiome data were a limited modality check

The multiome analysis included 42 donor-region observations from 14 donors across three regions; all 41 donors in the available multiome set overlapped with the singleome donor set. Its coefficient was negative but imprecise (*β* = −0.210, 95% CI: −0.908 to 0.488; *p* = 0.556; Supplementary Figure S2). This result does not constitute independent biological replication and provides only weak evidence that the direction is not unique to the singleome assay.

### Tau exposure lowered the detectable panel in cultured human microglia

Twelve of the 13 panel genes were available in the iPSC normalized-count matrix; HLA-DRB5 was absent. The expanded score was lower after fibrillar tau exposure than after vehicle-control exposure (difference = -1.343; exact allocation *p* = 0.029; Figures 3A,B). Eleven of the 12 gene-level fold changes were negative; HLA-DOB was close to zero and positive (Figure 3C). Monomeric tau and human AD-derived tau-versus-tau-depleted material also produced negative score differences (−0.964 and −1.208, respectively; Figure 3B; Supplementary Table S5). These seven-well comparisons support a tau-responsive transcriptional change but cannot establish whether the same process produced the postmortem association.

## Discussion

### Principal finding

The primary SEA-AD analysis links higher regional tau burden to lower pooled microglial HLA-II transcription within the same donors. The association was reproduced across three biologically motivated HLA-II panels and persisted after adjusting for local Aβ, RIN, NeuN-positive FANS fraction, and the abundance of a published DAM-enriched state. The much smaller GSE160936 analysis pointed in the same direction but did not survive its prespecified exact test and correction. The main evidential weight therefore rests on SEA-AD, with GSE160936 treated as supportive.

### How this fits with HLA-II-rich microglia near lesions

An inverse regional average does not negate the presence of HLA-II-rich cells near plaques or mixed lesions. Regional pseudobulk and lesion-proximal spatial analyses answer different questions. Kwon et al. (2023) reported lower expression of overlapping HLA-II genes in selected tau-associated spatial comparisons, while several spatial and proteomic studies have identified antigen-presenting myeloid niches around Aβ or mixed pathology (Wachter et al., 2024; Miyoshi et al., 2024; Mrdjen et al., 2025). Lu et al. (2026) placed an antigen-presentation-rich late program at an Aβ-tau inflection point, contrasting with an earlier complement/TREM2-rich program. Sampling scale, lesion mixture, region, disease stage, and cell-state definition can therefore yield coherent local enrichment and a lower region-wide average without either observation being erroneous.

### Composition and within-state expression

New state analyses narrow, but do not close, the interpretation. Micro-PVM_3-SEAAD abundance did not explain the pooled association, and a negative coefficient remained after adjustment. However, within-state estimates varied in precision, and the rare HLA-II-rich Micro-PVM_4-SEAAD state was not estimable under the donor threshold. The observed regional score can still combine changes in the abundance of unmeasured or finely resolved states with transcriptional shifts within states. We therefore do not describe the result as selective HLA-II suppression.

### Tau versus Aβ

The direction was more consistent for tau than for Aβ, but the formal coefficient contrast did not establish a difference. This is important because Aβ and tau covary across AD regions and may shape microglia sequentially or jointly. Our data support a tau-associated regional pattern after adjusting for measured Aβ; however, they do not establish tau specificity or exclude residual mixed-pathology effects.

### Human-cell context

Cultured human microglia data show that several tau preparations are associated with reduced HLA-II transcription over the experimental interval. This is an orthogonal observation, not a mechanistic validation. Seven wells limit precision, and the experiment cannot distinguish tau uptake from cellular stress, toxicity, preparation-related effects, or a change in cell state. It also does not recreate aging, chronic pathology, cellular interactions, or the anatomical gradients of the postmortem brain; tau–microglia relationships depend on experimental and phagocytic contexts (LeBlang et al., 2020).

### Strengths and limitations

The strengths of this study include a locked, comprehensive panel; explicit evidence of roles; donor-level inference; region-matched pathology; a model that separates within- from between-donor variation; region-, panel-, influence-, covariate-, composition-, and state-sensitivities; and transparent downgrading of small or overlapping datasets. Limitations remain substantial. Postmortem data are cross-sectional and subject to regional neuronal loss, tissue quality, unmeasured pathology, selection bias, and reverse causation. State annotations are imperfect, and sparse states could not be modeled. GSE160936 has only 11 paired donors; multiome donors overlap SEA-AD singleome donors, and the iPSC comparisons contain seven wells. HLA-II protein, peptide loading, functional antigen presentation, and T-cell interactions were not measured.

### Implications

The study identified a reproducible regional transcriptional association, with its significance depending on scale and cellular context. The most informative next experiment would combine continuous tau and Aβ measurements, lesion distance, microglial state, HLA-II RNA and protein levels, and functional peptide presentation across multiple regions from the same donors. Until then, the present results should be read as evidence about regional HLA-II transcription, rather than as proof that tau impairs antigen presentation.

## Conclusion

In the SEA-AD, regions with a greater tau burden compared to a donor’s sampled-region average had lower pooled microglial HLA-II transcription, and the association persisted across panel and measured covariate checks. A small paired cohort and cultured human microglia supplied directionally compatible observations but did not independently confirm the mechanism. State-composition analyses did not reduce the finding to one DAM-like population; however, neither tau specificity nor functional antigen presentation was established.

## Data Availability

Publicly available datasets were analyzed in this study. GSE160936 is available through the NCBI Gene Expression Omnibus. SEA-AD processed data and neuropathology metadata are available through the Allen Institute SEA-AD portal. The Karabova iPSC-microglia data and source analysis files are available through the repository linked by the source article. Aggregate revision outputs, the revision analysis code, and the locked analysis contract are supplied as Supplementary materials. The existing reproducibility record is available in Zenodo record 21717637 (https://doi.org/10.5281/zenodo.21717637). No person-level source data are redistributed.

## References

[ref1] Afify R. Lipsius K. Wyatt-Johnson S. J. Brutkiewicz R. R. (2024). Myeloid antigen-presenting cells in neurodegenerative diseases: a focus on classical and non-classical MHC molecules. Front. Neurosci. 18:1488382. doi: 10.3389/fnins.2024.1488382, 39720231 PMC11667120

[ref2] Avey D. R. Ng B. Vialle R. A. Kearns N. A. de Paiva Lopes K. Iatrou A. . (2025). Uncovering plaque-glia niches in human Alzheimer's disease brains using spatial transcriptomics. Mol. Neurodegener. Adv. 1:2. doi: 10.1186/s44477-025-00002-z, 40740481 PMC12310243

[ref3] Capitani A. Carpanini S. M. (2025). HLA'S hidden hand in Alzheimer's disease—five research questions en route to an answer. Brain Commun. 7:fcaf438. doi: 10.1093/braincomms/fcaf438, 41277898 PMC12631126

[ref4] Chhatbar C. Sankowski R. Schulz M. Shimizu T. Schwabenland M. Staszewski O. . (2026). A transcriptomic microglia taxonomy across mouse and human pathologies. Nat. Immunol. 27, 1066–1080. doi: 10.1038/s41590-026-02472-z, 41882205 PMC7618987

[ref5] Crowell H. L. Soneson C. Germain P. L. Calini D. Collin L. Raposo C. . (2020). Muscat detects subpopulation-specific state transitions from multi-sample multi-condition single-cell transcriptomics data. Nat. Commun. 11:6077. doi: 10.1038/s41467-020-19894-4, 33257685 PMC7705760

[ref6] Fixemer S. Miranda de la Maza M. Hammer G. P. Jeannelle F. Schreiner S. Gérardy J. J. . (2025). Microglia aggregates define distinct immune and neurodegenerative niches in Alzheimer's disease hippocampus. Acta Neuropathol. 149:19. doi: 10.1007/s00401-025-02857-8, 39954093 PMC11829914

[ref7] Gabitto M. I. Travaglini K. J. Rachleff V. M. Kaplan E. S. Long B. Ariza J. . (2024). Integrated multimodal cell atlas of Alzheimer's disease. Nat. Neurosci. 27, 2366–2383. doi: 10.1038/s41593-024-01774-5, 39402379 PMC11614693

[ref8] Karabova M. K. Del Ser-Badia A. Hedegaard A. Washer S. J. Baykam Z. O'Brien D. P. . (2026). Tracking tau and cellular responses in human iPSC-microglia: from uptake to seedable secretion, including in extracellular vesicles. Alzheimers Dement. 22:e71337. doi: 10.1002/alz.71337, 41943483 PMC13053939

[ref9] Keren-Shaul H. Spinrad A. Weiner A. Matcovitch-Natan O. Dvir-Szternfeld R. Ulland T. K. . (2017). A unique microglia type associated with restricting development of Alzheimer's disease. Cell 169, 1276–1290.e17. doi: 10.1016/j.cell.2017.05.018, 28602351

[ref10] Krasemann S. Madore C. Cialic R. Baufeld C. Calcagno N. El Fatimy R. . (2017). The TREM2-APOE pathway drives the transcriptional phenotype of dysfunctional microglia in neurodegenerative diseases. Immunity 47, 566–581.e9. doi: 10.1016/j.immuni.2017.08.008, 28930663 PMC5719893

[ref11] Kwon S. H. Parthiban S. Tippani M. Divecha H. R. Eagles N. J. Lobana J. S. . (2023). Influence of Alzheimer's disease related neuropathology on local microenvironment gene expression in the human inferior temporal cortex. GEN Biotechnol. 2, 399–417. doi: 10.1089/genbio.2023.0019, 39329069 PMC11426291

[ref12] LeBlang C. J. Medalla M. Nicoletti N. W. Hays E. C. Zhao J. Shattuck J. . (2020). Reduction of the RNA binding protein TIA1 exacerbates neuroinflammation in tauopathy. Front. Neurosci. 14:285. doi: 10.3389/fnins.2020.00285, 32327969 PMC7161592

[ref13] Lu A. Chen W. T. Dalby M. Sainz Garcia D. Vanheusden M. de Vries L. E. . (2026). Human microglial transitions at the aβ-tau inflection point associate with divergent pathways to dementia and resilience. Nat. Med. 32, 2047–2059. doi: 10.1038/s41591-026-04393-8, 42243549 PMC13278961

[ref14] Mancuso R. Fattorelli N. Martinez-Muriana A. Davis E. Wolfs L. Van Den Daele J. . (2024). Xenografted human microglia display diverse transcriptomic states in response to Alzheimer's disease-related amyloid-β pathology. Nat. Neurosci. 27, 886–900. doi: 10.1038/s41593-024-01600-y, 38539015 PMC11089003

[ref15] Martins-Ferreira R. Calafell-Segura J. Leal B. Rodríguez-Ubreva J. Martínez-Saez E. Mereu E. . (2025). The human microglia atlas (HuMicA) unravels changes in disease-associated microglia subsets across neurodegenerative conditions. Nat. Commun. 16:739. doi: 10.1038/s41467-025-56124-1, 39820004 PMC11739505

[ref16] Mary A. Mancuso R. Heneka M. T. (2024). Immune activation in Alzheimer disease. Annu. Rev. Immunol. 42, 585–613. doi: 10.1146/annurev-immunol-101921-035222, 38424470

[ref17] Masuda T. Sankowski R. Staszewski O. Böttcher C. Amann L. Sagar . (2019). Spatial and temporal heterogeneity of mouse and human microglia at single-cell resolution. Nature 566, 388–392. doi: 10.1038/s41586-019-0924-x, 30760929

[ref18] Mathys H. Boix C. A. Akay L. A. Xia Z. Davila-Velderrain J. Ng A. P. . (2024). Single-cell multiregion dissection of Alzheimer's disease. Nature 632, 858–868. doi: 10.1038/s41586-024-07606-7, 39048816 PMC11338834

[ref19] Mathys H. Davila-Velderrain J. Peng Z. Gao F. Mohammadi S. Young J. Z. . (2019). Single-cell transcriptomic analysis of Alzheimer's disease. Nature 570, 332–337. doi: 10.1038/s41586-019-1195-2, 31042697 PMC6865822

[ref20] Miyoshi E. Morabito S. Henningfield C. M. Das S. Rahimzadeh N. Shabestari S. K. . (2024). Spatial and single-nucleus transcriptomic analysis of genetic and sporadic forms of Alzheimer's disease. Nat. Genet. 56, 2704–2717. doi: 10.1038/s41588-024-01961-x, 39578645 PMC11631771

[ref21] Mrdjen D. Cannon B. J. Amouzgar M. Kim Y. Liu C. Vijayaragavan K. . (2025). Spatial proteomics of Alzheimer's disease-specific human microglial states. Nat. Immunol. 26, 1397–1410. doi: 10.1038/s41590-025-02203-w, 40696045 PMC13075585

[ref22] Paolicelli R. C. Sierra A. Stevens B. Tremblay M. E. Aguzzi A. Ajami B. . (2022). Microglia states and nomenclature: a field at its crossroads. Neuron 110, 3458–3483. doi: 10.1016/j.neuron.2022.10.020, 36327895 PMC9999291

[ref23] Prater K. E. Green K. J. Mamde S. Sun W. Cochoit A. Smith C. L. . (2023). Human microglia show unique transcriptional changes in Alzheimer's disease. Nat. Aging 3, 894–907. doi: 10.1038/s43587-023-00424-y, 37248328 PMC10353942

[ref24] Sanchez-Molina P. Rosmus D. D. Brownell D. Meral M. Gohlich C. Pratapa A. . (2026). Spatial proteomic analysis in human Alzheimer's disease brains enables identification of microenvironment-dependent microglial cell states. Nat. Neurosci. 29, 1599–1614. doi: 10.1038/s41593-026-02267-3, 42151483 PMC13337490

[ref25] Sankowski R. Böttcher C. Masuda T. Geirsdottir L. Sagar Sindram E. . (2019). Mapping microglia states in the human brain through the integration of high-dimensional techniques. Nat. Neurosci. 22, 2098–2110. doi: 10.1038/s41593-019-0532-y, 31740814

[ref26] Silvin A. Uderhardt S. Piot C. Da Mesquita S. Yang K. Geirsdottir L. . (2022). Dual ontogeny of disease-associated microglia and disease inflammatory macrophages in aging and neurodegeneration. Immunity 55, 1448–1465.e6. doi: 10.1016/j.immuni.2022.07.004, 35931085

[ref27] Smith A. M. Davey K. Tsartsalis S. Khozoie C. Fancy N. Tang S. S. . (2022). Diverse human astrocyte and microglial transcriptional responses to Alzheimer's pathology. Acta Neuropathol. 143, 75–91. doi: 10.1007/s00401-021-02372-6, 34767070 PMC8732962

[ref28] Squair J. W. Gautier M. Kathe C. Anderson M. A. James N. D. Hutson T. H. . (2021). Confronting false discoveries in single-cell differential expression. Nat. Commun. 12:5692. doi: 10.1038/s41467-021-25960-2, 34584091 PMC8479118

[ref29] Tuddenham J. F. Taga M. Haage V. Marshe V. S. Roostaei T. White C. . (2024). A cross-disease resource of living human microglia identifies disease-enriched subsets and tool compounds recapitulating microglial states. Nat. Neurosci. 27, 2521–2537. doi: 10.1038/s41593-024-01764-7, 39406950 PMC12094270

[ref30] Van Hove H. Martens L. Scheyltjens I. De Vlaminck K. Pombo Antunes A. R. De Prijck S. . (2019). A single-cell atlas of mouse brain macrophages reveals unique transcriptional identities shaped by ontogeny and tissue environment. Nat. Neurosci. 22, 1021–1035. doi: 10.1038/s41593-019-0393-4, 31061494

[ref31] Wachter A. Woodbury M. E. Lombardo S. Abdourahman A. Wuest C. McGlame E. . (2024). Landscape of brain myeloid cell transcriptome along the spatiotemporal progression of Alzheimer's disease reveals distinct sequential responses to aβ and tau. Acta Neuropathol. 147:65. doi: 10.1007/s00401-024-02704-2, 38557897 PMC10984903

[ref32] Wang L. P. Maxwell S. E. (2015). On disaggregating between-person and within-person effects with longitudinal data using multilevel models. Psychol. Methods 20, 63–83. doi: 10.1037/met0000030, 25822206

[ref33] Zimmerman K. D. Espeland M. A. Langefeld C. D. (2021). A practical solution to pseudoreplication bias in single-cell studies. Nat. Commun. 12:738. doi: 10.1038/s41467-021-21038-1, 33531494 PMC7854630

