# Supplementary material for: Regional tau burden tracks with lower microglial HLA class II transcription in the human brain
Source: Front Neurosci. 2026 Oct 7;20:1979981. doi: 10.3389/fnins.2026.1979981 (PMC13684807; doi:10.3389/fnins.2026.1979981)
Supplement: Supplementary file 1 [file Supplementary_file_1.docx]

**Supplementary Material**

# **Supplementary methods**

The revision analyses followed the locked contract dated 14 September 2026. Donors were resampled as intact clusters; nuclei, cells, regions, and modalities were not treated as independent people. Exact paired tests used all 2^11 sign flips. State scores used scaling constants from pooled resident-compatible pseudobulks within each assay so state-specific variance did not redefine the panel.

Supplementary Table S1. Locked HLA-II panel eligibility.

| **Gene** | **GSE detection** | **SEA-AD detection** | **Eligible** |
| --- | --- | --- | --- |
| CD74 | 1.000 | 1.000 | True |
| CIITA | 1.000 | 1.000 | True |
| HLA-DMA | 0.957 | 1.000 | True |
| HLA-DMB | 1.000 | 1.000 | True |
| HLA-DOA | 1.000 | 1.000 | True |
| HLA-DOB | 0.522 | 0.680 | True |
| HLA-DPA1 | 1.000 | 1.000 | True |
| HLA-DPB1 | 1.000 | 1.000 | True |
| HLA-DQA1 | 1.000 | 1.000 | True |
| HLA-DQB1 | 1.000 | 1.000 | True |
| HLA-DRA | 1.000 | 1.000 | True |
| HLA-DRB1 | 1.000 | 1.000 | True |
| HLA-DRB5 | 1.000 | 0.997 | True |

Supplementary Table S2. SEA-AD joint and covariate models.

| **Analysis** | **Term** | **β** | **CI low** | **CI high** | **P** |
| --- | --- | --- | --- | --- | --- |
| Joint tau + Aβ | Tau | -0.171 | -0.294 | -0.048 | 0.006 |
| Joint tau + Aβ | Aβ | -0.080 | -0.217 | 0.057 | 0.253 |
| Tau − Aβ contrast | Tau − Aβ | -0.091 | -0.279 | 0.097 | 0.343 |
| Covariate-adjusted | Tau | -0.167 | -0.289 | -0.045 | 0.007 |

Supplementary Table S3. State-composition and within-state analyses.

| **Analysis** | **State** | **Donors** | **β** | **CI low** | **CI high** | **P** | **Status** |
| --- | --- | --- | --- | --- | --- | --- | --- |
| DAM proportion | Micro-PVM_3 | 79.0 | 0.097 | -0.035 | 0.228 | 0.151 |  |
| HLA-II \| DAM | Micro-PVM_3 | 79.0 | -0.176 | -0.300 | -0.053 | 0.005 |  |
| Within state | Micro-PVM_2 | 77.0 | -0.139 | -0.269 | -0.010 | 0.035 | Fitted |
| Within state | Micro-PVM_2_1 |  |  |  |  |  | Not estimable |
| Within state | Micro-PVM_2_3 | 76.0 | -0.042 | -0.165 | 0.081 | 0.507 | Fitted |
| Within state | Micro-PVM_3 | 63.0 | -0.138 | -0.314 | 0.037 | 0.123 | Fitted |
| Within state | Micro-PVM_4 |  |  |  |  |  | Not estimable |

Abbreviation: HLA-II | DAM, HLA-II model additionally adjusted for the measured DAM-enriched-state proportion.

Supplementary Table S4. GSE160936 exact and influence-aware analyses.

| **Panel** | **Pathology** | **β** | **CI low** | **CI high** | **Exact P** | **BH q** | **Theil-Sen** | **Max Cook D** |
| --- | --- | --- | --- | --- | --- | --- | --- | --- |
| Expanded (13) | pTau | -0.533 | -0.923 | -0.143 | 0.083 | 0.166 | -0.396 | 0.291 |
| Expanded (13) | Aβ | 0.226 | -0.280 | 0.732 | 0.415 | 0.415 | 0.098 | 0.235 |
| Legacy (6) | pTau | -0.604 | -1.035 | -0.173 | 0.042 | 0.084 | -0.819 | 0.328 |
| Legacy (6) | Aβ | 0.205 | -0.361 | 0.771 | 0.472 | 0.472 | -0.047 | 0.206 |
| Lu late-PIG | pTau | -0.362 | -0.995 | 0.272 | 0.263 | 0.525 | -0.408 | 0.292 |
| Lu late-PIG | Aβ | 0.123 | -0.508 | 0.754 | 0.621 | 0.621 | 0.026 | 0.268 |

Supplementary Table S5. Expanded-panel iPSC-microglia contrasts.

| **Contrast** | **Treated** | **Control** | **Difference** | **Exact P** | **Genes** |
| --- | --- | --- | --- | --- | --- |
| Fibrillar tau vs vehicle | 4 | 3 | -1.343 | 0.029 | 12 |
| Monomeric tau vs vehicle | 4 | 3 | -0.964 | 0.029 | 12 |
| AD tau vs tau-depleted | 4 | 3 | -1.208 | 0.029 | 12 |

# **Supplementary Figure S1**

Supplementary Figure S1. HLA-II panel detection and published-state sensitivity. Micro-PVM_2_1-SEAAD and Micro-PVM_4-SEAAD did not meet the prespecified 20-donor threshold.

# **Supplementary Figure S2**

Supplementary Figure S2. Influence and modality sensitivity. (A) Cook's distances for individual GSE160936 donors; the dashed line is the common 4/n reference. (B) SEA-AD multiome estimate. All 14 multiome donors overlapped singleome donors, so this is not independent biological replication.

# **Supplementary Figure S3**

Supplementary Figure S3. Regional pathology distributions in SEA-AD. Points are medians and bars are interquartile ranges for local AT8-positive area and local Aβ.
